# Diffusion-weighted magnetic resonance imaging for predicting the clinical outcome of comatose survivors after cardiac arrest: a cohort study

**DOI:** 10.1186/cc8874

**Published:** 2010-02-12

**Authors:** Seung Pill Choi, Kyu Nam Park, Hae Kwan Park, Jee Young Kim, Chun Song Youn, Kook Jin Ahn, Hyeon Woo Yim

**Affiliations:** 1 Department of Emergency Medicine, College of Medicine, The Catholic University of Korea, 505 Banpo-dong, Seocho-gu, Seoul, 137-701, Korea; 2 Department of Neurosurgery, College of Medicine, The Catholic University of Korea, 505 Banpo-dong, Seocho-gu, Seoul, 137-701, Korea; 3 Department of Radiology, College of Medicine, The Catholic University of Korea, 505 Banpo-dong, Seocho-gu, Seoul, 137-701, Korea; 4 Clinical Research Coordinating Center, Departments of Preventive Medicine, College of Medicine, The Catholic University of Korea, 505 Banpo-dong, Seocho-gu, Seoul, 137-701, Korea

## Abstract

**Introduction:**

The aim of this study was to examine whether the patterns of diffusion-weighted imaging (DWI) abnormalities and quantitative regional apparent diffusion coefficient (ADC) values can predict the clinical outcome of comatose patients following cardiac arrest.

**Methods:**

Thirty-nine patients resuscitated from out-of-hospital cardiac arrest were prospectively investigated. Within five days of resuscitation, axial DWIs were obtained and ADC maps were generated using two 1.5-T magnetic resonance scanners. The neurological outcomes of the patients were assessed using the Glasgow Outcome Scale (GOS) score at three months after the cardiac arrest. The brain injuries were categorised into four patterns: normal, isolated cortical injury, isolated deep grey nuclei injury, and mixed injuries (cortex and deep grey nuclei). Twenty-three subjects with normal DWIs served as controls. The ADC and percent ADC values (the ADC percentage as compared to the control data from the corresponding region) were obtained in various regions of the brains. We analysed the differences between the favourable (GOS score 4 to 5) and unfavourable (GOS score 1 to 3) groups with regard to clinical data, the DWI abnormalities, and the ADC and percent ADC values.

**Results:**

The restricted diffusion abnormalities in the cerebral cortex, caudate nucleus, putamen and thalamus were significantly different between the favourable (n = 13) and unfavourable (n = 26) outcome groups. The cortical pattern of injury was seen in one patient (3%), the deep grey nuclei pattern in three patients (8%), the cortex and deep grey nuclei pattern in 21 patients (54%), and normal DWI findings in 14 patients (36%). The cortex and deep grey nuclei pattern was significantly associated with the unfavourable outcome (20 patients with unfavourable vs. 1 patient with favourable outcomes, *P *< 0.001). In the 22 patients with quantitative ADC analyses, severely reduced ADCs were noted in the unfavourable outcome group. The optimal cutoffs for the mean ADC and the percent ADC values determined by receiver operating characteristic (ROC) curve analysis in the cortex, caudate nucleus, putamen, and thalamus predicted the unfavourable outcome with sensitivities of 67 to 93% and a specificity of 100%.

**Conclusions:**

The patterns of brain injury in early diffusion-weighted imaging (DWI) (less than or equal to five days after resuscitation) and the quantitative measurement of regional ADC may be useful for predicting the clinical outcome of comatose patients after cardiac arrest.

## Introduction

Although advances in cardiopulmonary resuscitation and critical care medicine have considerably increased the chances of patient survival after cardiac arrest, most of these patients suffer ischemic brain injury and often remain comatose for some time [1]. The degree of cerebral damage must be determined as early as possible to plan and administer appropriate post-resuscitation therapy and to support the counseling of family members, but it is often difficult to achieve with certainty [2]. Various methods have been assessed for predicting the neurological outcome of comatose survivors after cardiac arrest, including clinical examination, electroencephalogram, somatosensory evoked potentials (SSEPs), and biochemical markers. However, despite improvements in early prognostic evaluation, there are still some limitations and defects to solve, such as clinical examination and electroencephalogram being difficult to apply under sedative treatment [3], SSEPs having a moderate sensitivity in spite of 100% specificity for the prediction of persistent coma [4], and biochemical markers being susceptible to false positive results [5].

Neuroimaging, such as computed tomography (CT) scans or magnetic resonance imaging (MRI), is useful in assessing the extent of structural brain injury. Yet, evaluating hypoxic ischemic brain injury with CT or conventional MRI often underestimates the actual extent of injury in the acute period [6,7]. In contrast to CT and conventional MRI, diffusion-weighted imaging (DWI) can reveal the acute or early subacute findings following a focal ischemic stroke or global cerebral hypoxia [7,8], and this technique allows quantitative assessment of the severity of brain damage by means of measuring the apparent diffusion coefficient (ADC) [9-12]. 

The patterns and extent of brain injury seen in DWI are associated with clinical outcomes in neonates with perinatal asphyxia [13] and patients after cardiac arrest [14,15]. DWI abnormalities in large areas including the cerebral cortex, basal ganglia, and cerebellum suggest devastating diffuse hypoxic ischemic necrosis, whereas a pattern of DWI abnormality restricted to the basal ganglia or selected cortical regions suggests mild hypoxic injury. For a patient stricken with an acute ischemic stroke, the severity of the neuronal injury within a lesion seen by DWI reflects the degree of apparent diffusion coefficient (ADC) alteration [16]. The ADCs of cortex and basal ganglia measured during the early life (≤ six days) of neonates suffering with perinatal asphyxia has also been reported to correlate with the late prognosis [17]. The high cortical signal of DWI with a marked ADC decrease in the early phase of global cerebral hypoxia correlates with irreversible tissue injury or cortical laminar necrosis, and it may be an early marker of the clinical outcome [14,15,18,19]. Recently, two studies reported quantitative ADC analyses of the whole brain or regional brain as a significant prognostic tool for predicting poor outcome in comatose survivors after cardiac arrest [20,21].

Therefore, the purpose of our study was to examine whether the patterns of DWI abnormalities and regional ADC values by a regions-of-interest (ROIs)-based method can predict the clinical outcome of comatose patients following cardiac arrest.

## Materials and methods

### Subjects

This study was reviewed and approved by the local ethics committee of our university hospital. Between January 2004 and December 2007, we prospectively studied 39 patients at St. Mary's Hospital (a tertiary-care university hospital in Seoul, Korea) who survived an out-of-hospital cardiac arrest. We included the adult patients (≥ 18 years) who were successfully resuscitated from the cardiac arrest, survived for at least 24 h, and remained comatose for at least 6 h after return of spontaneous circulation (ROSC) to avoid transient unconsciousness. The exclusion criteria included cardiac arrest resulting from intracranial haemorrhage, drug intoxication, trauma or a terminal illness, a previous history of neurological disease or brain trauma, a lack of informed consent, and being unavailable for follow-up. The study group included 28 men and 11 women (mean age: 49.1 years, range: 18 to 89 years) (Table 1).

**Table 1:** The clinical characteristics of the 39 comatose patients who were resuscitated from cardiac arrest

	Favourable outcome	Unfavourable outcome	*P *value
Patients (n)	13	26	
Age (y)	50.0 ± 16.2	48.5 ± 18.5	0.810
Gender (male/female)	8/5	20/6	0.314
Witnessed arrest (n)	8	16	1.000
Bystander CPR (n)	8	15	0.818
Initial ECG on admission (n)			0.008
PEA	0	5	
VF/pulseless VT	5	1	
Asystole	8	20	
Resuscitation duration (min)	14.8 ± 9.7	15.4 ± 12.4	0.892
Time to MRI after ROSC (h)	54.3 ± 44.5	52.2 ± 35.3	0.872
Cause of arrest (n)			0.077
Cardiac	8	8	
Respiratory	3	16	
Unknown	2	2	
GOS (n)			
1. Death	0	10	
2. Vegetative state	0	14	
3. Severe neurologic impairment	0	2	
4. Mild to moderate neurologic impairment	2	0	
5. Complete recovery	11	0	

Mean ± S.D.; CPR, cardiopulmonary resuscitation; ECG, electrocardiogram; PEA, pulseless electrical activity; VF, ventricular fibrillation; VT, ventricular tachycardia; ROSC, return of spontaneous circulation; GOS, Glasgow outcome scale

Gender, witnessed arrest, bystander CPR, initial ECG on admission and cause of arrest in both groups were analysed by Chi-squared test or Fisher's exact test.

Age, resuscitation duration and time between MRI and ROSC in both groups were compared by *t*-test.

The patients were evaluated in terms of age, gender, cause of death, if the collapse was witnessed, if a bystander performed cardiopulmonary resuscitation (CPR), the initial electrocardiogram (ECG) on admission, the duration of resuscitation, the Glasgow coma scale (GCS) score within 6 h after ROSC, the time between MRI and ROSC, and the Glasgow outcome scale (GOS) score [22]. The resuscitation protocols followed the American Heart Association guidelines [23,24]. If intracranial haemorrhage was suspected, brain CT was examined as soon as possible after resuscitation. All of the patients were admitted to an intensive care unit (ICU), and they received standard intensive care and monitoring, including mechanical ventilation, arterial catheters, central venous catheters, urinary catheters, and rectal temperature measurements. Neurological examinations were performed at zero, six hours, one day, three days, five days, one week and two weeks after cardiac arrest. SSEPs were performed between one and three days after ROSC. A standardised protocol for therapeutic hypothermia was used in comatose patients during the latter half of the study period. Eligible patients underwent therapeutic hypothermia using an external cooling device for 24 h with a target temperature of 33.0 ± 1°C. Slow rewarming to normal temperature was conducted over eight hours. In patients with therapeutic hypothermia, MRI was performed after normothermia. All of the patients underwent limited MRI that was confined to a DWI and a T2-weighted image (T2WI) for rapid image acquisition (<10 minutes) within five days after resuscitation (the acute and early subacute phases) while avoiding pseudonormalisation of the DWI [15]. The neurological outcomes of the patients were assessed using the GOS score at three months after the cardiac arrest. There was no withdrawal of life support. The comatose patients were divided into two groups: the GOS scores between 1 and 3 (death, vegetative state, and severe disability) were grouped as unfavourable outcomes; and the GOS scores of 4 and 5 (moderate disability and good recovery) were grouped as favourable outcomes. The control group consisted of 16 men and 7 women (mean age: 51.7 years, range: 30 to 80 years) who were examined and scanned at the emergency department for dizziness; they were free of neurological disorders or brain trauma with normal brain MRIs. Informed consent was obtained from the patients' relatives and all controls.

### Magnetic resonance imaging

In total, 22 of the 39 patients and 23 control subjects were assessed using a 1.5-T system (Signa Excite; General Electric, Milwaukee, WI, USA) that had echo planar capability. These studies included the following sequences: the axial fast spin-echo T2WIs (4000/1002/2 [TR/TE/NEX] with a 5 mm section thickness) and the axial DWIs (7000/105.2 [TR/TE], a section thickness 5 mm, b values of 0 and 1,000 sec/mm^2^, a field of view 240 × 240, and a matrix size 128 × 128). The other 17 patients were examined with a 1.5-T system (Magnetom Vision Plus; Siemens, Erlangen, Germany) that had echo planar capability, with the following sequences: the axial fast spin-echo T2WIs (4500/99/2 [TR/TE/NEX] with a 5 mm section thickness), and the axial DWIs (5700/139 [TR/TE], a section thickness 5 mm, b values of 0 and 1,000 sec/mm^2^, a field of view 240 × 240, and a matrix size 96 × 128). The ADC maps were automatically generated. Only the DWI data and ADC maps were analysed for this study. The T2WI was used to detect old hyperintense abnormalities to exclude chronic infarction, or it was used as a reference image for this study. The MRIs were reviewed on a standard picture archiving and communication system workstation (Maroview; Marotech, Seoul, Korea). The DWIs together with the ADC maps of all the comatose patients were jointly evaluated by two experienced neuroradiologists blinded to the patients' clinical data. The brain injuries on DWI were categorised into four patterns on the basis of the injury region of the grey matter: normal, isolated cortical injury, isolated deep grey nuclei injury (including caudate nucleus, putamen, and thalamus), and mixed cortical and deep grey nuclei injuries.

**Figure 1:**
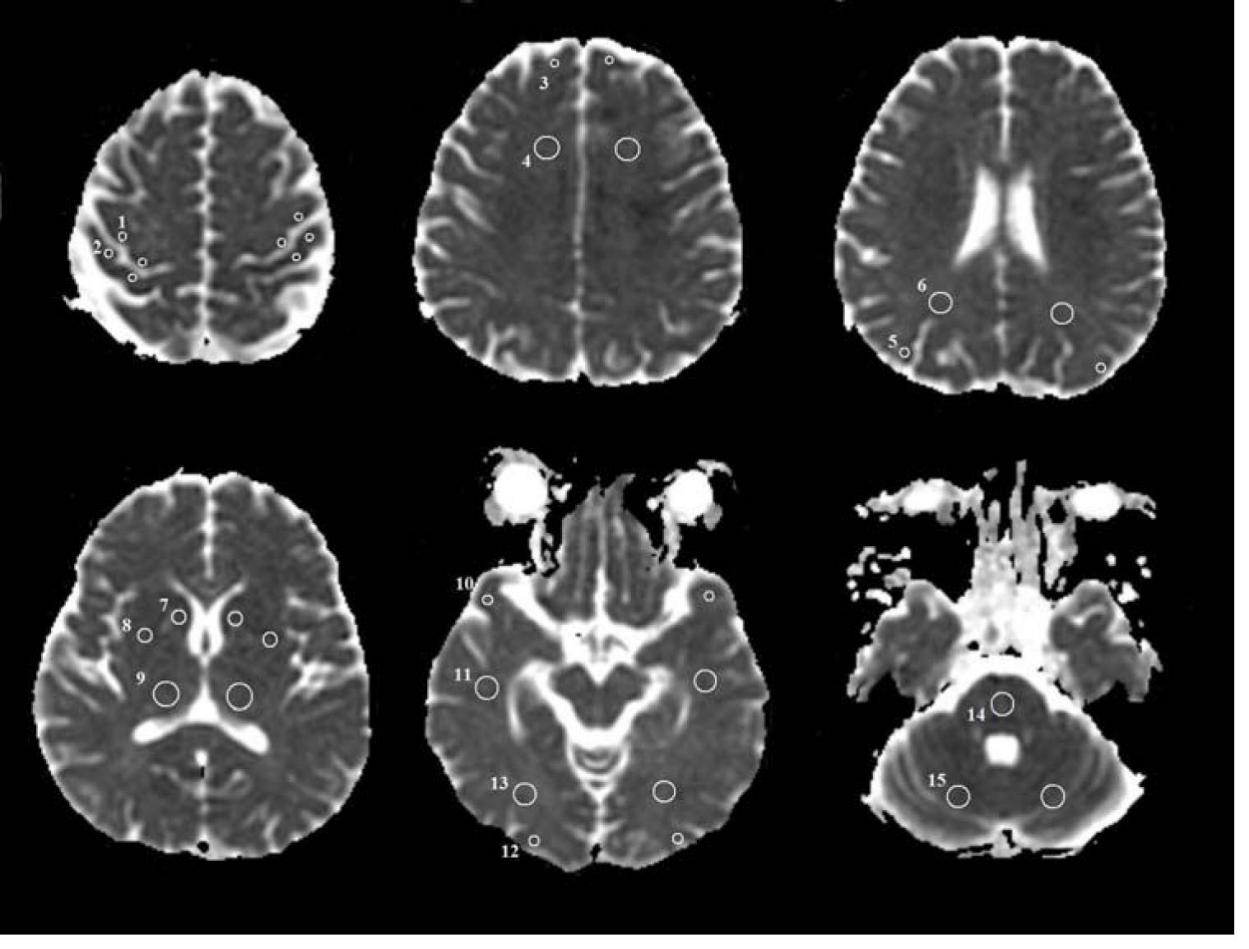
**This figure shows the axial apparent diffusion coefficient maps indicating the 15 regions of interest**. These regions were selected for quantitative measurement of the apparent diffusion coefficient values. (1) precentral cortex, (2) postcentral cortex, (3) frontal cortex, (4) frontal white matter, (5) parietal cortex, (6) parietal white matter, (7) caudate nucleus, (8) putamen, (9) thalamus, (10) temporal cortex, (11) temporal white matter, (12) occipital cortex, (13) occipital white matter, (14) pons, and (15) cerebellum.

**Figure 2:**
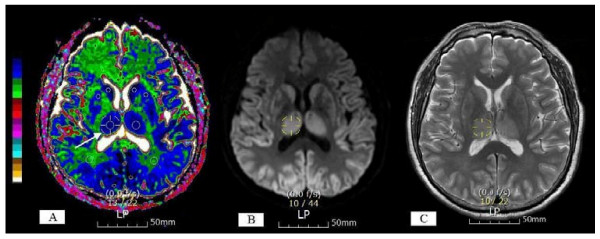
**Apparent diffusion coefficient map with colour shades (A), diffusion-weighted imaging (B) and T2-weighted image (C) from one representative patient at seven hours after cardiac arrest**. Regions of low apparent diffusion coefficient (ADC) showed a blue colour; in contrast, regions of high ADC showed a white colour. The colour shades on the ADC maps identified the pixel showing the minimum ADC value in each brain region. A 3D cursor (arrow) was used to select the predefined spot (right thalamus) simultaneously in the three different sequences, and it can be easy to mark the area with the minimum ADC on the ADC maps based on the T2-weighted image (T2WI) and diffusion-weighted imaging (DWI). The circular region-of-interest (ROI) cursors were positioned on the areas with the minimum ADC in each brain region. Severely restricted diffusion within the ROIs was shown in the caudate nucleus (0.238 × 10^-3^mm^2^/sec), putamen (0.299 × 10^-3^mm^2^/sec), thalamus (0.290 × 10^-3^mm^2^/sec), and occipital grey matter (0.184 × 10^-3^mm^2^/sec) but not in the occipital white matter (0.712 × 10^-3^mm^2^/sec).

The ADC values were only obtained from 22 patients who were examined using the GE Signa Excite due to the use of two kinds of MR scanners. On the workstation, the ADC value of each pixel was constantly displayed on the screen with a movement of a region of interest (ROI) cursor. For each patient, the region of a high signal on the DWI and a low signal on the ADC map was identified. The ROIs were positioned on the areas with a minimum ADC on the ADC maps to produce ADC values for each brain region. If the brain regions were normal, then the ROIs were positioned on the predefined locations (Figure 1). The colour shades were used on the ADC maps to visualise the degree of ADC decrease. Regions of low ADC showed a blue colour; in contrast, regions of high ADC showed a white colour (Figure 2). The colour shades on the ADC maps identified the pixel showing the minimum ADC value in each brain region. The ADC measurements from both sides of the brain were averaged as a patient's ADC value or a control ADC value. ROI sizes varied by region, using 4 mm^2 ^for cortex, 10 mm^2 ^for the caudate nucleus and putamen and 25 to 40 mm^2 ^for the subcortical white matter, thalamus, cerebellum, and pons. The percentage of the patient's ADC, as compared to the average normal control ADC in 15 different brain regions, was computed as a percent ADC value. The person placing the ROIs was blinded to the patient's outcome. To ensure accurate localisation and consistency of the measurements, the ROIs were carefully placed by a single analyst (SPC) who worked in consultation with a neuroradiologist who had 15 years of experience reading MRIs.

### Statistical analyses

The data were expressed as means ± standard deviations. Chi-squared and Fisher's exact tests were used to assess qualitative data (clinical: gender, witnessed arrest, bystander CPR, initial ECG on admission, and cause of arrest; MRI: abnormalities of each region of brain). *T*-tests were used to compare the quantitative data (clinical: age, resuscitation duration, and time between MRI and ROSC). A one-way ANOVA with the Scheffe post hoc test was applied to study the ADC values in the different regions of the brain. Box and whisker plots were constructed to summarise the distributions of the percent ADC values for the control, the favourable and the unfavourable outcome groups. Spearman's correlation test was used to correlate GOS at three months after ROSC with ADC values of each brain region. Correlations between ADC values of each brain region used a Pearson's correlation. Sensitivity, specificity, positive predictive values (PPV), and negative predictive values (NPV) for predicting unfavourable outcome were calculated using the optimal cutoff values determined by ROC curve analysis. The cutoff level predicting unfavourable outcome with 100% specificity was considered to be optimal. A *P *value of < 0.05 was considered significant. Statistical analysis was performed using the Statistical Package for Social Sciences (SPSS) version 15.0 (SPSS Inc., Chicago, IL, USA).

## Results

### General characteristics

During the study period, 240 patients unrelated to trauma suffered out-of-hospital cardiac arrest with attempted resuscitation. Of them, 131 achieved ROSC for more than 20 minutes, and 89 patients were admitted to the hospital alive. Sixty-five patients remained comatose for at least 6 h and survived for more than 24 h after admission. Of the 65 patients, 26 were excluded from this study as follows: lack of MRI data because of early death before MRI (n = 10); MRI delay over five days after ROSC (n = 3); no informed consent (n = 4); cardiac arrest due to intracranial haemorrhage (n = 12); and previous history of Parkinson's disease (n = 1), brain operation (n = 2), or cerebral infarction (n = 1). Thus, 39 were included in this study; 13 patients were assigned to the favourable outcome group (GOS 4 to 5), and 26 were assigned to the unfavourable outcome group (GOS 1 to 3). The mean time to MRI after ROSC for the patients was 52.9 ± 37.5 hours (range, 6 to 119 hours). The clinical characteristics of the patients are summarised in Table 1. There were no significant differences between the two groups, except for the initial ECG rhythm on admission. The mean duration in the intensive care was 11.5 ± 7.6 days (range, 3 to 31 days) in patients with favourable outcome and 21.1 ± 19.6 days (range, 2 to 91 days) in patients with unfavourable outcome. Ten patients (25.6%) died with a mean survival period of 9.2 ± 8.0 days (range, 2 to 29 days). Therapeutic hypothermia was performed in 15 (38%) of 39 analysed patients and 4 of the 15 patients had a favourable outcome. Myoclonic or seizure activities were seen within the first three days after ROSC in 15 patients (38.4%). Of 15 patients, 10 had an unfavourable outcome. Pupillary light reflex was often seen within first three days in patients with both favourable and unfavourable outcome. Eleven patients (28%) who showed loss of pupillary light reflex had an unfavourable outcome. Motor response to pain was absent at three days after cardiac arrest in 15 patients (38%) who had an unfavourable outcome. For 20 (51%) of 39 patients, CT scans were performed within 3 h after the event, and the scans were read as normal for 15 patients. The CT scans of the other five patients were interpreted as having brain edema, and they had an unfavourable outcome. In 20 (51%) of 39 patients, SSEP was examined between one and three days after cardiac arrest. Of them, eight patients who showed no cortical response had an unfavourable outcome.

### Qualitative analysis of the DWI

The cortex and basal ganglia were frequently damaged in the patients but predominantly in the unfavourable outcome group (81% vs. 8%, *P *< 0.001; and 77% vs. 23%, *P *= 0.002, respectively). In terms of cortical injuries, the Rolandic (precentral and postcentral), occipital, and parietal cortices had more frequent injury than did the frontal and temporal cortices. The cerebellum and pons had no differences in DWI abnormalities between the favourable and unfavourable outcome groups. The subcortical white matter had no DWI abnormality in any of the patients (Table 2). The neurological outcome in relation to DWI patterns is shown in Table 3. The cortical pattern of injury was seen in one patient (3%), the deep grey nuclei pattern was seen in three patients (8%), the cortex and deep grey nuclei pattern was seen in 21 patients (54%), and normal DWI findings were seen in 14 (36%). There were significant differences in the number of patients with normal findings or mixed cortex and deep grey nuclei injuries between the two groups (Fisher's exact test, *P *< 0.001). However, the cortical pattern and the deep grey nuclei pattern had no difference in the clinical outcome between the two groups.

**Table 2:** Restricted diffusion abnormalities on the diffusion-weighted imaging for patients with anoxic encephalopathy

Brain region	Favourable outcome (n = 13)	Unfavourable outcome (n = 26)	*P *value
Cerebral cortex	1 (8)	21 (81)	< 0.001
Frontal	0 (0)	18 (69)	< 0.001
Parietal	1 (8)	20 (77)	< 0.001
Temporal	1 (8)	16 (62)	0.002
Occipital	1 (8)	20 (77)	< 0.001
Rolandic	1 (8)	21 (81)	< 0.001
Precentral	0 (0)	21 (81)	< 0.001
Postcentral	1 (8)	20 (77)	< 0.001
Subcortical white matter	0 (0)	0 (0)	
Basal ganglia	3 (23)	19 (77)	0.002
Caudate nuclei	2 (15)	17 (65)	0.006
Putamen	2 (15)	17 (65)	0.006
Thalamus	2 (15)	16 (62)	0.008
Cerebellum	0 (0)	7 (27)	0.073
Pons	0 (0)	2 (8)	0.544

The data are given in numbers (percentages) of patients. Statistical analyses were done by Fisher's exact test.

**Table 3:** Patterns of diffusion-weighted imaging abnormalities in the two outcome groups

Pattern	Favourable outcome (n = 13)	Unfavourable outcome (n = 26)
Normal*	10 (77)	4 (15)
Cortex	0 (0)	1 (7)
Deep grey nuclei	2 (15)	1 (7)
Cortex and deep grey nuclei*	1 (8)	20 (77)

The data are given in numbers (percentages) of patients.

Deep grey nuclei include caudate nucleus, putamen, and thalamus in this study.

*There were significant differences (Fisher's exact test, *P *< 0.001) in the number of patients with normal findings or mixed cortex and deep grey nuclei injuries between the two groups. Other comparisons were non-significant between the groups.

### Quantitative analysis of the ADC values

#### ROI analysis

**Figure 3:**
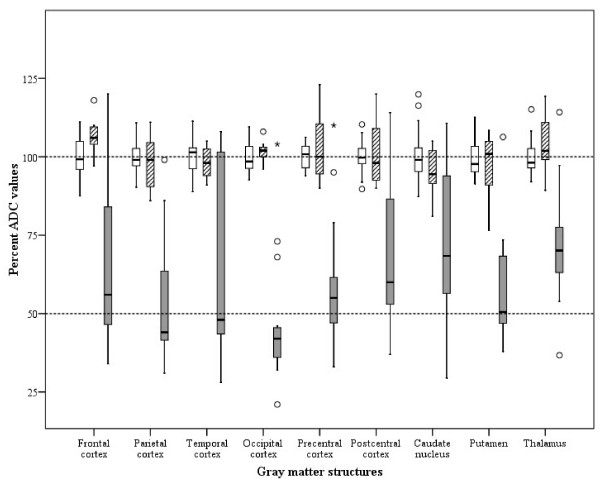
**Boxplot showing the distribution of the percent apparent diffusion coefficient values for the different brain regions of the control (white bars), favourable (striped bars), and unfavourable (grey bars) groups**. The percent apparent diffusion coefficient (ADC) values were calculated using the mean normal control value of each brain region.

The ADC value was measured in 22 patients: 8 had a favourable outcome, and 14 had an unfavourable outcome. Among the grey matter structures of 22 patients, the precentral cortex showed the lowest mean ADC value (0.598 ± 0.234 × 10^-3 ^mm^2^/sec), whereas the temporal cortex had the highest mean ADC value (0.710 ± 0.277 × 10^-3 ^mm^2^/sec). In all regions, the mean ADC values of the favourable outcome group were similar to those of the controls. The favourable outcome group had significantly different mean ADC values and percent ADC values than the unfavourable outcome group in the frontal, parietal, temporal, occipital, precentral, and postcentral cortices, the caudate nucleus, the putamen, and the thalamus (Table 4) (Figure 3) (*P *< 0.05). The unfavourable outcome group had significantly different mean ADC values than the controls in the frontal, parietal, temporal, occipital, precentral and postcentral cortices, the frontal white matter, the caudate nucleus, the putamen, and the thalamus (*P *< 0.05).

**Table 4:** The ADC values of the individual brain regions in the patients and the control subjects (mean ADC ± SD; × 10^-3^mm^2^/sec)

Brain region	Patients (n = 22)		Controls (n = 23)
		
	Favourable outcome (n = 7)	Unfavourable outcome (n = 15)	
Frontal cortex	0.917 ± 0.056	0.563 ± 0.232^a,b^	0.859 ± 0.057
Frontal white matter	0.736 ± 0.031	0.738 ± 0.050^a^	0.697 ± 0.035
Parietal cortex	0.860 ± 0.088	0.475 ± 0.167^a,b^	0.877 ± 0.040
Parietal white matter	0.741 ± 0.070	0.774 ± 0.084	0.744 ± 0.043
Temporal cortex	0.910 ± 0.052	0.616 ± 0.291^a,b^	0.926 ± 0.050
Temporal white matter	0.781 ± 0.044	0.796 ± 0.056	0.778 ± 0.026
Occipital cortex	0.911 ± 0.032	0.417 ± 0.184^a,b^	0.896 ± 0.044
Occipital white matter	0.768 ± 0.086	0.741 ± 0.067	0.762 ± 0.032
Precentral cortex	0.743 ± 0.085	0.425 ± 0.149 ^a,b^	0.719 ± 0.028
Postcentral cortex	0.737 ± 0.083	0.494 ± 0.173^a,b^	0.725 ± 0.036
Caudate nucleus	0.770 ± 0.069	0.589 ± 0.204^a,b^	0.808 ± 0.065
Putamen	0.763 ± 0.093	0.456 ± 0.138^a,b^	0.788 ± 0.050
Thalamus	0.824 ± 0.079	0.563 ± 0.144^a,b^	0.790 ± 0.044
Cerebellum	0.731 ± 0.084	0.690 ± 0.130	0.757 ± 0.043
Pons	0.740 ± 0.072	0.753 ± 0.111	0.769 ± 0.056

^a^Significant compared to the controls at *P *< 0.05 using one way analysis of variance (ANOVA) with the Scheffe post hoc test.

^b^Significant when comparing the unfavourable outcomes to the favourable outcomes at *P *< 0.05 using one way ANOVA with the Scheffe post hoc test.

ADC, apparent diffusion coefficient

#### Relation between GOS and ADC values

Correlation coefficients between ADC values in each cortex, caudate nucleus, putamen, and thalamus were large (Pearson's correlation, r = 0.559-0.925; all *P *< 0.001). Considering the relationship between the GOS score and the ADC values of the grey matter structures, Spearman's correlation coefficients for the GOS score vs. the mean ADC values were: frontal cortex: r_s _= 0.544, *P *= 0.009; parietal cortex: r_s _= 0.702, *P *< 0.001; occipital cortex: r_s _= 0.782, *P *< 0.001; precentral cortex: r_s _= 0.597, *P *= 0.003; postcentral cortex: r_s _= 0.515, *P *= 0.014; caudate nucleus: r_s _= 0.470, *P *= 0.027; putamen: r_s _= 0.746, *P *< 0.001; and thalamus: r_s _= 0.731, *P *< 0.001. Among the grey matter structures, high correlations between the GOS score and the mean ADC values were observed in the occipital and parietal cortices, putamen, and thalamus (all r_s _> 0.7, all *P *< 0.001).

#### Sensitivity, specificity, PPV, and NPV of mean ADC variables in predicting unfavourable outcome

In order to predict the unfavourable outcome, the optimal cutoffs for the mean ADC and the percent ADC values in the grey matter structures were derived from the ROC curve analysis (Table 5). The areas under the ROC curve were greater than 0.9 for ADC values in the parietal, occipital and precentral cortices, putamen, and thalamus (all *P *< 0.001). The optimal cutoffs for the mean ADC and the percent ADC values in each cortex, caudate nucleus, putamen, and thalamus predicted the unfavourable outcome with sensitivities of 67 to 93% and a specificity of 100%. In particular, the cutoffs of the occipital cortex and putamen produced the highest accuracy (Table 5).

**Table 5:** Prediction of unfavourable outcome using the optimal cutoffs of the ADC and the percent ADC

Grey matter structures	Optimal cutoff		Sensitivity with 95% CI	Specificity with 95% CI	PPV with 95% CI	NPV with 95% CI
					
	ADC	Percent ADC**				
Frontal cortex	0.685	79	73% (45 to 85%)	100% (56 to 100%)	100% (68 to 100%)	64% (32 to 88%)
Parietal cortex*	0.674	77	87% (58 to 98%)	100% (56 to 100%)	100% (72 to 100%)	78% (40 to 96%)
Temporal cortex	0.640	69	67% (39 to 87%)	100% (56 to 100%)	100% (66 to 100%)	58% (29 to 84%)
Occipital cortex*	0.740	82	93% (66 to 100%)	100% (56 to 100%)	100% (73 to 100%)	88% (47 to 99%)
Precentral cortex*	0.606	84	87% (58 to 98%)	100% (56 to 100%)	100% (72 to 100%)	78% (40 to 96%)
Postcentral cortex	0.625	86	73% (45 to 91%)	100% (56 to 100%)	100% (68 to 100%)	64% (32 to 88%)
Caudate nucleus	0.621	76	67% (39 to 87%)	100% (56 to 100%)	100% (66 to 100%)	58% (29 to 84%)
Putamen*	0.590	75	93% (66 to 100%)	100% (56 to 100%)	100% (73 to 100%)	88% (47 to 99%)
Thalamus*	0.647	85	87% (58 to 98%)	100% (56 to 100%)	100% (72 to 100%)	78% (40 to 96%)

ADC unit × 10^-3 ^mm^2^/sec; PPV, positive predictive value; NPV, negative predictive value

Optimal cutoff values predicting unfavourable outcome were determined by ROC curve analysis in patients and controls.

*Area under the ROC curve was greater than 0.9 with a *P *value less than 0.001.

**Percent ADC expressed the (absolute ADC value/mean normal control ADC value) × 100.

## Discussion

The results of this study suggest that the pattern of brain injury on early DWI (≤ five days after resuscitation) and quantitative measurements of regional ADC may help predict the clinical outcome of comatose patients after cardiac arrest. Conventional MRI is not a helpful prognostic tool in the early phase after global cerebral hypoxia because it may reveal normal or only subtle abnormality [7,15]. Conversely, DWI could give prognostic values for comatose patients because it is very sensitive for detecting cerebral ischemia [14,15,18,19]. DWI provides an approximation of the water motion in brain tissue. In early anoxic encephalopathy, a dysfunction of the membrane bound Na-K-ATPase pump is caused by ischemia and this leads to a shift of water from the extracellular compartment to the intracellular compartment, which restricts intracellular water motion [25,26]. This restricted diffusion is markedly hyperintense on DWI. DWI can show the restricted diffusion associated with acute ischemia 30 minutes after a witnessed ictus in the patients with acute stroke. The ADC is most reduced at 8 to 32 h and remains markedly reduced for three to five days [26]. Therefore, DWI may be of greater diagnostic utility to detect cerebral ischemia within five days after the event [15,18].

Findings of this study have shown that different patterns of brain injury relate to clinical outcome. Diffusion abnormality of the cortex was mainly observed in the unfavourable outcome group. Most of the patients with cortical abnormalities also had combined deep grey nuclei abnormalities. Thus, the mixed pattern of injury (cortex and deep grey nuclei) often showed diffuse and bilateral abnormalities and seems to correlate with the most severe brain injury of postcardiac arrest survivors [14,15]. Therefore, the mixed pattern of injury was most predictive of an unfavourable outcome, although one patient, whose DWI showed subtle abnormalities in the cortex and basal ganglia, had a good neurological recovery in this study. On the other hand, a normal finding of DWI indicated a high probability of a favourable outcome. Among 14 patients with normal DWI findings, four patients had an unfavourable outcome. One of these four patients died due to massive haemoptysis during the ICU stay. Another patient suffered from chronic renal failure before the cardiac arrest, which contributed to the unfavourable outcome. However, the two patients did not have any specific cause having an unfavourable outcome, suggesting that the normal finding of DWI is not always associated with a favourable outcome [17,27,28].

Concerning the location of cortical injury, the Rolandic, parietal, and occipital cortices were more frequently injured than were the frontal and temporal cortices, which is consistent with findings in previous studies [14,27]. This result suggests that the Rolandic, parietal, and occipital cortices are most affected by global cerebral hypoxia. In the Rolandic cortex, many net-associated pyramidal cells predominantly populate layers III and V, which are vulnerable to hypoxia [29]. The occipital lobe and the precuneus are known to be supplied by the posterior cerebral artery and partly by the anterior cerebral artery, and these arteries intermingle for anastomosis in the medial parietal lobe. For both arteries, the occipital lobe and the precuneus are the last border zone of the brain artery network [30]. Therefore, hypoxic ischemic injuries may specifically induce neuronal death in these areas.

In this study, the ROIs were not positioned in the same location for all the patients and were located in the visually abnormal areas seen on DWI. This may have induced significant bias because the normal ADC values are not homogeneous in the different regions of the brain. However, Helenius et al. [31] demonstrated in a study of 80 healthy volunteers that the ADC values alone were not site-specific, and no differences were found in the various cortical grey matter and white matter regions. Therefore, although the ROIs in this study are not positioned in the same location of brain, the ADC value for each region can be thought to be a representative value for each patient. The reported normal ADC values in the grey matter and white matter were 0.78 to 1.09 × 10^-3 ^mm^2^/sec and 0.62 to 0.79 × 10^-3 ^mm^2^/sec, respectively [31]. These values are similar to our control ADC values. However, the control ADC values in the Rolandic cortex (0.65 to 0.80 × 10^-3 ^mm^2^/sec) were lower than those in the other cortices and were similar to those in the subcortical white matter, which might be explained by the low signal intensity in the perirolandic cortex of the normal brain on the T2WI and the fluid attenuated inversion recovery (FLAIR) images due to the histologic background [32,33].

The high cortical signal on DWI during the early phase of global cerebral hypoxia correlates with irreversible tissue injury or cortical laminar necrosis. Kawahara et al. [34] reported that DWI showed hyperintensity in the cerebral cortex of vegetative patients on Day 3, and laminar hyperintensity was observed in the same area on the T1-weighted images on Day 14. Thus, DWI can be very useful for detecting cortical laminar necrosis in patients with anoxic hypoxic encephalopathy in the early subacute phase (one to five days) [19]. Lovblad et al. [19] demonstrated that in 19 patients with cortical laminar infarcts, the ADC value decreased to 60 to 80% of the normal value in the bilateral or localised cortical lesions seen on DWI, and all of the patients were dead or survived with severe disabilities. Els et al. [14] also reported that in 9 of 12 patients with global cerebral hypoxia, the ADC values of the cortex were reduced to 60 to 80% of the normal value on DWI within 36 h after cardiac arrest, and this led to a vegetative state after six months. In our study, the ADC values in the grey matter structures (including the cortex and deep grey nuclei) with restricted diffusion decreased to 21 to 79% of that of the controls, and although the percent ADC values had a wide range, the upper value was approximately 80% of normal, which was similar to that of the previous studies. In a small study of six patients with extremely poor outcomes [18], all of them showed a mean ADC value of 0.35 × 10^-3 ^mm^2^/sec in the precentral cortex in the early phase (one to five days) after a severe anoxic event, which was comparable with the mean ADC value of 0.42 × 10^-3 ^mm^2^/sec in the unfavourable outcome group of this study. Thus, ADC values of the grey matter structures decreased to less than 80% of normal may indicate a cortical laminar necrosis or an irreversible tissue injury and this may well predict an unfavourable outcome.

The degree of the changes of the DWI and the ADC signal intensity correlates with the severity of neuronal injury because modest changes reflect signs of ongoing lesions, and a severe drop of the ADC corresponds to cell death [10]. In this study, high correlations were observed between the GOS and the ADC values of the parietal and occipital cortices, putamen, and thalamus. The extent of the DWI abnormalities that occurs with the ADC decrease is of importance to determine the outcome of patients [14]. Recently, two studies [20,21] evaluated the extent of DWI abnormalities by measuring whole brain ADC values and the predicted clinical outcome of patients after cardiac arrest. Wu et al. [20] demonstrated in 80 comatose patients with cardiac arrest that a whole-brain median ADC less than 0.665 × 10^-3 ^mm^2^/sec was a significant predictor of poor outcome based on no eye opening or a six month modified Rankin scale score greater than 3. Wijiman et al. [21] reported that the percentage (10% cutoff value) of brain volume below the ADC threshold of 0.650 × 10^-3 ^mm^2^/sec differentiated between survivors and patients who died or remained vegetative, whereas mean brain ADC values did not differentiate between outcome groups in contradiction to Wu et al.'s results. However, in the setting of hypoxic ischemic encephalopathy following cardiac arrest, for a patient who has global cerebral injury that is generally widespread, the severity of the injury may be expressed by the degree of the altered ADC value in any specific area (for example, the parietal and occipital cortices, putamen, and thalamus) in the early phase. Therefore, we believe that on the DWI performed within five days of anoxic encephalopathy, if there is a mixed pattern of injury (cortex and the deep grey nuclei) and if the ADC value in any grey matter is reduced to less than 80%, then this may allow us to predict an unfavourable outcome.

There are several limitations of this study. First, two different scanners were used for the patients, and a smaller number of patients than all of the study patients were used to determine the cutoff value of the ADC for predicting an unfavourable outcome. Thus, a larger number of patients are needed to confirm this. Second, ROI-based analysis was done on the confined areas that showed diffusion restriction. If the patients had segmental infarction with a low ADC in the confined area, this may produce a bias for predicting clinical outcome. Yet, all patients in this study did not have any segmental infarction. In 22 patients with ADC measurement, 6 had normal DWI findings, 14 had a bilateral injury of the cortex and deep grey nuclei, and 2 had a bilateral putamen injury. Third, partial volume averaging of the subcortical white matter, which has a lower ADC value than the grey matter, would be expected to reduce the measured ADC values of the grey matter. Fourth, although MRI was performed within five days after ROSC to avoid pseudonormalisation of the DWI, the MRIs were taken at different times, and this could have influenced the ADC changes due to the evolution of the abnormality seen on DWI [7,28]. Fifth, this study included patients with or without induced hypothermia, which did not statistically influence patient outcome. We cannot expect an effect of induced hypothermia on a brain's ADC abnormality. Sixth, the intensivists who treated the patients were not kept blinded from the MRI data of the patients, and this data was used for counseling the patients' families, although there was no withdrawal of life support. Thus, this could have produced a bias in the patients' treatment by the intensivists.

## Conclusions

Our study has revealed that the mixed pattern of brain injury (the cortex and deep grey nuclei) on DWI performed within five days after cardiac arrest is well-correlated with an unfavourable outcome. The recognition of brain injury pattern using DWI may be important to determine clinical outcome of the comatose patients after out-of-hospital cardiac arrest. In addition, there was a relationship between the GOS and the regional ADC values of the grey matter structures, in which cutoffs of ADC values were helpful in discriminating an unfavourable from a favourable outcome. Therefore, the pattern of brain injury and quantitative measurement of regional ADC may predict the clinical outcome of comatose patients following their cardiac arrest.

## Key messages

• Diffusion-weighted imaging is an important diagnostic method for predicting the clinical outcome of comatose survivors after out-of-hospital cardiac arrest.

• The cortex and basal ganglia were predominantly damaged in the patients, and in particular, the Rolandic, parietal, and occipital cortices were most frequently injured in the patients with an unfavourable outcome.

• The mixed pattern of brain injury (including the cortex and deep grey nuclei) on DWI in the early phase (less than or equal to five days) of anoxic encephalopathy was well-correlated with an unfavourable outcome three months after out-of-hospital cardiac arrest.

• The relationship between the GOS and the regional ADC values of the cortex and deep grey nuclei was observed, and cutoffs of ADC values discriminated between an unfavourable and a favourable outcome.

## Abbreviations

ADC: apparent diffusion coefficient; CPR: cardiopulmonary resuscitation; CT: computed tomography; DWI: diffusion-weighted imaging; ECG: electrocardiogram; EEG: electroencephalogram; FLAIR: fluid attenuated inversion recovery; GCS: Glasgow coma scale; GOS: Glasgow outcome scale; ICU: intensive care unit; MRI: magnetic resonance imaging; NPV: negative predictive values; PPV: positive predictive values; ROC: receiver operating characteristic; ROI: region of interest; ROSC: return of spontaneous circulation; SSEP: somatosensory evoked potential; T2WI: T2-weighted image.

## Competing interests

The authors declare that they have no competing interests.

## Authors' contributions

SPC participated in data collection, analysis and interpretation, and drafted the manuscript. KNP conceived the study, participated in its design and coordination and helped to draft the manuscript. HKP collected data. JYK collected and interpreted radiologic data. CSY collected data and helped with the study design. KJA collected and interpreted radiologic data. HWY participated in the study design and performed the statistical analyses. All authors read and approved the final manuscript.
